# Effects of drought stress on water content and biomass distribution in summer maize(*Zea mays* L.)

**DOI:** 10.3389/fpls.2023.1118131

**Published:** 2023-03-02

**Authors:** Siying Yan, Baisha Weng, Lanshu Jing, Wuxia Bi

**Affiliations:** ^1^ State Key Laboratory of Simulation and Regulation of Water Cycle in River Basin, China Institute of Water Resources and Hydropower Research, Beijing, China; ^2^ Yinshanbeilu Grassland Eco-hydrology National Observation and Research Station, China Institute of Water Resources and Hydropower Research, Beijing, China; ^3^ College of Hydrology and Water Resources, Hohai University, Nanjing, China

**Keywords:** drought, water content, biomass, allometric growth, correlation

## Abstract

The resource allocation of different component organs of crops under drought stress is a strategy for the coordinated growth of crops, which also reflects the adaptability of crops to drought condition. In this study, maize variety namely ‘Denghai 618’, under the ventilation shed, two treatment groups of light drought (LD) and moderate drought (MD), and the same rehydration after drought are set, as well as the normal water supply for control in shed (CS). The drought experiment was conducted in the jointing–tasseling stage in 2021. The effects of different drought stress on the water content and biomass allocation of each component organ were analyzed. The results showed that (1) during the drought period, the water content of each component organ of summer maize decreased in general, but the Water content distribution ratio (WCDR) of the root increased by 1.83%– 2.35%. The WCDR of stem increased by 0.52%– 1.40%. (2) Under different drought treatments, the root biomass (RB) increased 33.94% ~ 46.09%, and fruit biomass (FB) increased 1.46% ~ 2.49%, the leaf biomass (LB) decreased by 8.2% and 1.46% respectively under LD and MD. (3) The allometric growth model constructed under sufficient water is not suitable for drought stress; the allometric exponent α under drought stress is lower than that of the CS: CS (α=1.175) > MD (α = 1.136) > LD (α = 1.048), which also indicates that the impact of existing climate change on grain yield may be underestimated. This study is helpful to understand the adaptive strategies of the coordinated growth of maize component organs under drought stress and provide a reference for the prediction of grain yield under climate change.

## 1 Introduction

With the impact of global climate change and intensified human activities, most of the world’s land is affected by drought, which is one of the biggest factors affecting crop growth and development (Osmolovskaya et al., 2018; Kaukab and Sowbiya, 2021; Zhang et al., 2022). The yield of maize, as the main food crop in the world, is mainly limited by drought (Saini and Westgate, 2000; Zhou et al., 2011). Understanding the adaptability of maize under drought stress is crucial to the growth, development, and yield of maize, which can ensure food security under the background of climate change (Egamberdiyeva, 2007; Sposito, 2013). At present, many scholars (Mubeen et al., 2021; Salehi et al., 2021; Li et al., 2022; Muroyiwa et al., 2022) have carried out a lot of research on the impact of drought on maize, but few of them (Yan et al., 2022b) pay attention to the adaptability of maize under drought stress, especially the adaptability of the coordinated growth of maize component organs under drought (Song and Dai, 2005), which limits the understanding of crop adaptation strategies in arid environments.

Crops adapt to drought through a series of physiological, morphological, and biochemical processes (Mahdid et al., 2011; Huseynova et al., 2016; Mubeen et al., 2021; Prince et al., 2022). Water is not only an important component of crops but also the raw material and medium of most life activities (Wei et al., 2016; David et al., 2018; Yang et al., 2022). The ability of crops to maintain an appropriate water status and effectively use available resources is crucial for their growth and survival in water- deficient environments (Shao et al., 2005). Generally speaking, the root is an important organ for crops to supplement nutrition and absorb water (Grzesiak et al., 2014; Yan et al., 2022a). Its main function is to fix and support the plant, absorb water and minerals dissolved in water, transport water and minerals to the stem, and store nutrients (Smith and Smet, 2012). The stem is the main transport organ, which can transport nutrients and water and prop up leaves, flowers, and fruits in a definite space (Shang et al., 2020; Zhang et al., 2021). The leaf is the most important organ for the photosynthesis of plants and has transpiration function, providing the power to absorb water and mineral nutrients from the outside from the root (Liu et al., 2022; Suryanarayanan et al., 2022). The fruit is a reproductive organ, which plays a role in propagation and reproduction. Its material accumulation after maturity also reflects the crop yield (Isabel et al., 2022). The water content of each component organ of a plant reflects its metabolic activity (Pan, 2014). Different component organs of the same plant have different water content. Generally, the component organs with vigorous metabolism have a high water content (Wang, 2010; Tao et al., 2020). The distribution of water among roots, stems, leaves, and fruits is an important resource allocation form of crops, which can directly reflect the pattern of water acquisition and utilization among various components of crops (Garbin and Dillenburg, 2009). Some studies have shown that (Luo et al., 2020), under water stress, the water content of different component organs is different, and their resource allocation rates are different, indicating that plants can adjust their own balance through internal redistribution.

The accumulation of biomass in crops reflects the ability to use resources and the environment (Jevon and Lang, 2022). The biomass allocation of each component organ of crops is considered as one of the important strategies for plants to adapt to stress. Many studies have proven that (Pandit et al., 2020), under drought stress, many plants can effectively improve their adaptability to drought stress by adjusting their own material distribution pattern. For instance, under drought stress, crops will preferentially accumulate the biomass of roots to improve the water absorption capacity and reduce the amount of dry matter in stems and leaves above ground to reduce transpiration and water loss, thus improving the overall adaptability of plants (Liu et al., 2004). The drought adaptation mechanism of reducing the biomass distribution of aboveground parts by increasing the root is a common drought adaptation strategy of plants, which reflects the cooperative growth strategy among component organs.

This experiment is mainly done to deeply understand the adaptability of coordinated growth among the component organs of summer maize under drought stress. Different degrees of drought were set in the field, and the effects of different drought stress on the water content and biomass distribution component organs were analyzed. The hypotheses of this study are as follows: (1) drought stress would make different component organs affected to different degrees and affect the water content distribution ratio (WCDR) of summer maize; (2) drought stress would affect the distribution of biomass, and the allometric growth model established is different from the control in shed (CS); and (3) there is a relationship between the water content and biomass of component organs in summer maize. Under drought stress, how to adjust the distribution of the water content and biomass of maize to adapt to this stress is a problem that cannot be ignored. Determining the coordinated growth of maize component organs during drought will help to understand the adaptive strategies of maize and provide a basis for targeted drought control measures.

## 2 Materials and methods

### 2.1 Overview of the experimental area

The experimental site was selected at the Wudaogou Hydrological Experimental Station (33°09′N, 117°21′E) in Anhui province, China, which is located in the northern Anhui plain and is the main crop- producing area. The crop layout is mainly arid crops, and the farming system is mostly double cropping a year. The average annual precipitation for many years is 899.2 mm, of which the precipitation during the growth period of summer maize (June to October) accounts for approximately 68.66% (**Supplementary Table 1**). The soil type is Shajiang black soil, which is characterized by poor permeability, easy drought and waterlogging, low organic matter content, the lack of phosphorus and nitrogen, the soil texture being too sticky, and the plough sole being thick. The return period of drought in this area is 2–3 years, and summer is the growing season of maize in this area. Moreover, maize is not only the major crop in the northern Anhui plain but also the dominant crop in this area.

### 2.2 Experimental setup

**Figure 1:**
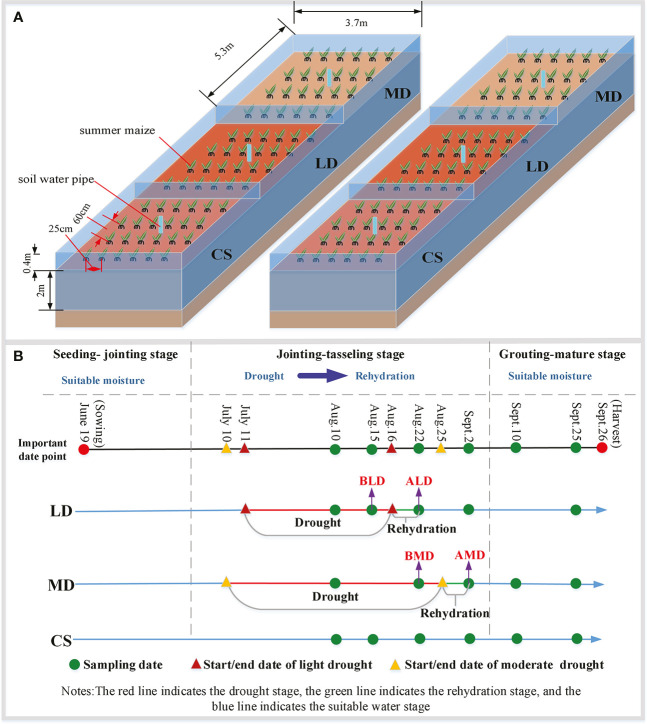
Design diagram of the experimental scheme. **(A)** is the design diagram of the experimental field; **(B)** is a simplified diagram of drought- level experimental design.

In 2021, the control experiment of controlling the water content was carried out during the jointing– tasseling stage. A total of six control experimental fields were set up to observe the growth law of summer maize under different drought scenarios. Due to the limited number of experimental fields, each group is provided with two repeated experimental fields, but the measured data can ensure three or more repetitions. The size of each experimental field is 5.3 m × 3.7 m; there have been a partition around the field to block the lateral seepage and cross flow between the fields. The depth of the partition in the underground part is 2 m, and the above ground part is 0.4 m higher than the ground level. One soil moisture aluminum tube was buried in each experimental field to measure the soil moisture of summer maize at different depths. In this study, the maize variety is normal variety ‘Denghai 618’. Before sowing, the experimental field needs to be applied with the same amount of fertilizer, based on 22,500 kg km^-2^ urea and 75,000 kg km^-2^ maize compound fertilizer. The soil water content was controlled to reach the same level 5 days before sowing, and the soil water content was measured the day before sowing. The summer maize in the field is 6.74 plants m^-2^, the row spacing of summer maize in the field is 60 cm, and the plant spacing is 25 cm (**Figure 1A**).

In the jointing–tasseling stage in 2021, the experiments of light drought, moderate drought, and rehydration after drought and the control group without drought inside the shed were set up (**Figure 1B** **)**. The LD and MD were sampled five times, and the CS was sampled six times (green point in **Figure 1B**). Among them, the data used for LD mentioned below refer to the data sampled on August 15, denoted by BLD, and the data used for rehydration after LD (RLD) refer to the data sampled on August 22, denoted by ALD. The data used for MD mentioned below refer to the data sampled on August 22, denoted by BMD, and the data used for rehydration after MD (RMD) refer to the data sampled on September 2, denoted by AMD.

The drought grade standard in this study is divided by the relative humidity of 10–20 cm soil layer in the Meteorological Drought Grade (GB/T20481-2006, 2006) **(** **Table 1** **)**. The calculation formula of soil relative humidity is shown in formula (1).

**Table 1:** Classification standard of drought grade.

Grade	Relative humidity of soil at the depth of 10–20 cm
No drought	60%< R
Light drought	50%< R ≤ 60%
Moderate drought	40%< R ≤ 50%
Severe drought	30%< R ≤ 40%
Extreme drought	R ≤ 30%


(1) $$R=\frac{w}{f}\times 100\%$$

where R is the relative humidity of soil (%); w is the soil water content (%); f is the soil field capacity (%); according to the test of the experimental station for many years, the soil field water capacity is approximately 30.7%.

### 2.3 Measurement and calculation of items

#### 2.3.1 Measurement items

##### 2.3.1.1 Soil moisture content

In the experiment, the soil water content was measured each day with an AIM-WIFI soil multiparameter monitoring system (Beijing AoZuo Ecology Instrumentation Ltd., Beijing, China). The soil measurement accuracy of the instrument was ± 2%, and the measurement repetition accuracy was ± 0.3%. During the test, the soil moisture content was measured every morning, 7:00–9:00 a.m. (GMT+8). The soil relative humidity data are calculated from the soil moisture content of 10 cm layer of field. In the test, the measurement is repeated for three times, and the test data are taken as the average value of three times.

##### 2.3.1.2 Plant sample measurement

In this subject, in order to observe the change characteristics of growth morphology during the growth period of summer maize and determine the biomass of various organs of summer maize, first, dig the whole maize plant with a shovel. Four maize plants are dug in each treatment group (two plants each field). Then, wash the soil on the roots, wipe out the water stains, separate and sort out the various organs of maize, weigh the fresh weight of each component organ, dry them in the drying box (the temperature is set to 105 °C), and then weigh them. Finally, get the dry weight (also called biomass).

#### 2.3.2 Calculation items

(1) The water content of the component organ is calculated as formula (2),


(2) $$W{∁}_{i}=\frac{W_{i}-W_{i}^{'}}{W_{i}}\times 100$$

where *WC_i_* is the water content of a component organ; *W_i_* is the fresh weight of a component organ; $W_{i}^{'}$ is the dry weight of the same component organ; and *i* refers to the root, stem, leaf, and fruit.

(2) The WCDR of the component organ is calculated by formula (3),


(3) $$DR_{i}=\frac{W_{i}-W_{i}^{'}}{\sum^{}W_{i}-\sum^{}W_{i}^{'}}\times 100$$

Where *DR_i_* is the WCDR of component organ; Σ*W_i_* is the fresh weight of the whole plant; $\sum W_{i}^{'}$ is the dry weight of the whole plant; *W_i_* is the fresh weight of a component organ; $W_{i}^{'}$ is the dry weight of the same component organ; and *i* refers to the root, stem, leaf, and fruit.

(3) The change rate of the water content refers to the change amount of the water content per unit time; the formula for the change rate of the water content is calculated as formula (4),


(4) $$A_{i}=\frac{WC_{i+D}^{'}-WC_{i}^{'}}{D}$$

where *A_i_* is the change rate of the water content of a component organ (unit: %/d); WC_i_’ is the water content of a component organ at time; *WC’*_*i*+*D*_ is the water content of the same component organ after *D* days; *D* is the time interval (unit: days); and *i* refers to the root, stem, leaf, and fruit.

(4) The proportion of the biomass of a component organ is calculated as formula (5),


(5) $$P_{i}=\frac{W_{i}^{'}}{\sum^{}W_{i}^{'}}$$

where, *P_i_* is the proportion of the biomass of a component organ; $\sum W_{i}^{'}$ is the dry weight of the whole plant; $W_{i}^{'}$ is the dry weight of a component organ; *i* refers to the root, stem, leaf, and fruit.

(5) The root– shoot ratio is calculated by formula (6),


(6) $$R_{a}=\frac{W_{r}^{'}}{\sum W_{i}^{'}-W_{r}^{'}}$$

where *R_a_* is the root– shoot ratio; $W_{r}^{'}$ is the root dry weight; and ${\sum W}_{i}^{'}$ is the dry weight of the whole plant.

#### 2.3.3 Allometric growth model

In order to reveal the law of summer maize, we introduced the allometric growth model with underground biomass and aboveground biomass as the research object, and its formula (Weiner et al., 2009) is shown in formula (7). After the logarithmic conversion of data to homogenize variance, scaling exponents (slope) and allometric constants (intercept) are determined through linear regression, as shown in formula (8),


(7) $$Y=\beta X^{\alpha }$$

(8) logY=logβ+αlogX

where X is the underground biomass (also root biomass); Y is the aboveground biomass; *β* is often referred to as the allometric coefficient, and log *β* is called intercept; *α* is the allometric exponent or the slope, where α = 1 is isokinetic growth, α>1 is positive allometric growth, and α<1 is negative allometric growth.

### 2.4 Statistical analysis

The obtained data were statistically calculated and processed by Microsoft Excel 2010, and the data were analyzed by one-way ANOVA, LSD, and Pearson methods with the IBM SPSS statistics 26 (α = 0.05). The experimental data results are expressed as average values, and the graph is drawn in Origin2018.

## 3 Results

### 3.1 Water content distribution

#### 3.1.1 Water content under different drought conditions

**Figure 2:**
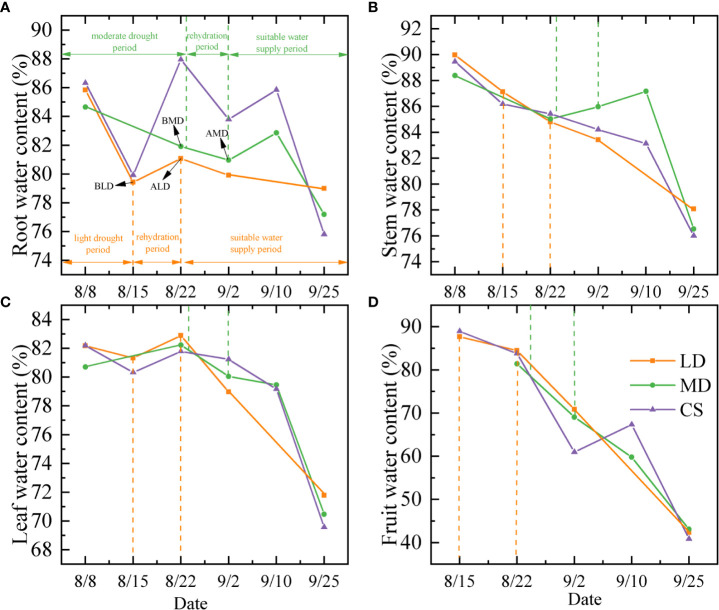
Analysis on dynamic changes of the water content in component organs of summer maize under different drought conditions. **(A–D)** are the dynamic changes of the water content in the root, stem, leaf, and fruit of summer maize, respectively. LD, MD, and CS represent the light drought treatment group, moderate drought treatment group, and control treatment group, respectively.

The dynamic change of the water content in each component organ of summer maize under different drought conditions is shown in **Figure 2**. The root water content (RWC) of summer maize decreased first, then increased, and then decreased with time **(** **Figure 2A** **)**. Under LD and MD (refers to BLD and BMD, the same below), the RWC was 79.44% and 81.91%, it was 0.5% and 6.04% lower than the CS, respectively, but there was no significant difference. The rate of decrease of RWC during LD was 0.91%/d **(** **Table 2** **)**, and the RWC increased by 1.65% (increased rate = 0.24%/d) in RLD (refers to ALD, the same below). The rate of the decrease of RWC during MD was 0.2%/d; the RWC did not increase in RMD (refers to AMD, the same below), with a decreased rate of 0.09%/d.

**Table 2:** Analysis on the rate of increase (decrease) of the water content of component organs under different periods.

Period	LD period	RLD period	MD period	RMD period
Rate of RWC (%/d)	-0.91	0.24	-0.20	-0.09
Rate of SWC (%/d)	-0.41	-0.33	-0.24	0.09
Rate of LWC (%/d)	-0.12	0.22	0.11	-0.20

The LD period, RLD period, MD period, and RMD period represent the light drought period, the period of rehydration after light drought, the moderate drought period, and the period of rehydration after moderate drought, respectively. The rate of RWC, SWC, and LWC represents the increase or decrease rate of the water content in the root, stem, and leaf.

The stem water content (SWC) showed a decreased trend in the whole measurement period **(** **Figure 2B** **).** In LD and MD, the SWC was 87.13% and 85.03%, which had no significant difference with the CS at the same period. The rate of decreased of SWC was 0.41%/d and 0.24%/d in the LD and MD period, respectively. In RLD, the SWC still decreased by 2.34% (decreased rate = 0.24%/d). The SWC increased in RMD. It increased by 0.94% (increased rate = 0.09%/d).

The leaf water content (LWC) showed a decreased trend in the whole measurement period **(** **Figure 2C** **).** The change was not very obvious before the date September 10 and then decreased rapidly. The LWC was 81.33% and 82.23% in LD and MD, which were 1.01% and 0.44% higher than that of the CS, respectively, but there was no significant difference. The rate of decrease of LWC during LD was 0.12%/d, and the rate of increase of LWC during MD was 0.11%/d. The LWC increased by 1.55% (increased rate = 0.22%/d) in RLD. The LWC decreased by 2.17% (decreased rate = 0.2%/d).

The fruit water content (FWC) showed a decreasing trend in the whole measurement period **(** **Figure 2D** **).** The FWC was 87.68% and 81.44% under the LD and MD, which was 1.28% and 2.37% lower than that of the CS, respectively. The FWC after rehydration is still declining, and the FWC in RLD and RMD is 3.19% and 12.36% lower than that in drought.

#### 3.1.2 Comparative analysis of component organs’ water content

**Figure 3:**
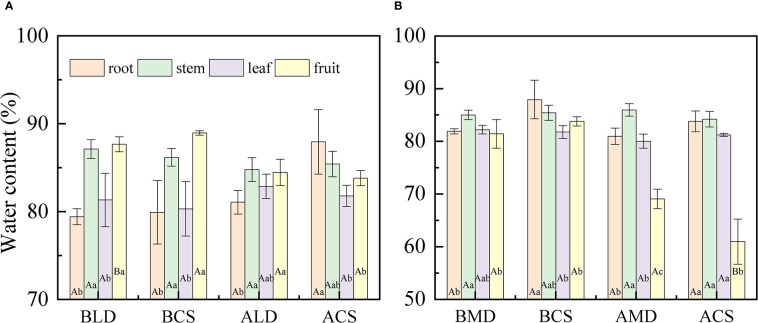
Comparative analysis of the water content of each component organ under different drought conditions and after rehydration. **(A, B)** are the comparative analysis of the water content of each component organ in light drought and moderate drought, respectively. Error bars represent standard deviations (n = 3), different capital letters indicate significant difference between groups (p< 0.05), and different lowercase letters indicate significant difference between different component organs of the same group (p< 0.05). BLD, BCS, ALD, and ACS represent before light drought, control group in the same period during light drought, after rehydration in light drought, and control group in the same period during after rehydration, respectively. Similarly, BMD, BCS, AMD, and ACS represent before moderate drought, control group in the same period during moderate drought, after rehydration in moderate drought, and control group in the same period during after rehydration, respectively.

Through a comparative analysis of the water content of each component organ drought and after rehydration **(** **Figure 3** **)**, it was found that there was a significant difference in the water content of the component organs of summer maize, and the difference changed with the growth period. When LD occurs, the water content of each component organ is as follows **(** **Figure 3A** **)**: fruit (87.68%) > stem (87.13%) > leaf (81.33%) > root (79.44%). The SWC and FWC were significantly higher than that of the RWC and LWC, which was similar to the CS at the same time. In RLD, the difference between component organs decreased, only the SWC and FWC were significantly higher than the RWC, and there was no significant difference between other component organs. In MD, the water content of each component organ is that **(** **Figure 3B** **)** stem (85.03%) > leaf (82.23%) > root (81.91%) > fruit (81.44%), and the SWC was significantly higher than the RWC and FWC. Different from the MD, the relationship of the water content of each component organ in the CS is as follows: root (87.95%) > stem (85.43%) > fruit (83.81%) > leaf (81.79%). The RWC was significantly higher than that of the LWC and FWC. In RMD, because this period is the fruit- ripening stage (fruit dehydration), the FWC sharply decreases, which was significantly lower than that of other component organs. The CS also has similar differences.

#### 3.1.3 Component organ water content distribution ratio

**Figure 4:**
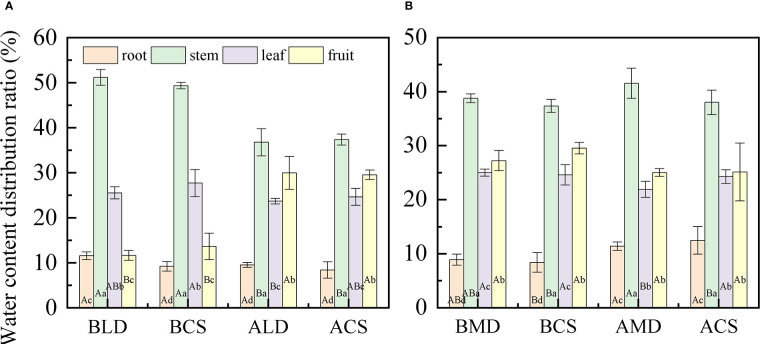
Comparative analysis of the WCDR of component organs in different drought and after rehydration. **(A, B)** are comparative analysis of the WCDR of component organs in LD and MD, respectively. Error bars represent standard deviations (n = 3), different capital letters indicate significant difference between groups (p< 0.05), and different lowercase letters indicate significant difference between different organs in the same group (p< 0.05). Note: BLD, BCS, ALD, and ACS and BMD, BCS, AMD, and ACS have the same definitionsas in **Figure 3**.

The WCDR of each component organ of summer maize under different drought stress is shown in **Figure 4**, calculated by formula (3). The WCDR of the root in different treatment groups was the lowest, ranging from 8.42% to 18.4%, and the WCDR of the stem was the highest, ranging from 34.31% to 51.2%. No matter under LD or MD, the WCDR of the root was higher than the CS in the same period, while the WCDR of the fruit was lower than the CS in the same period, but there was no significant difference. In LD **(** **Figure 4A** **)**, the WCDR of each component organ of summer maize was as follows: stem (51.2%) > leaf (25.54%) > fruit (11.67%) > root (11.59%). The WCDR of the stem was significantly higher than that of other component organs, and the CS had a similar distribution law at the same period. In RLD, the WCDR of the root, stem and leaf decreases, while the WCDR of the fruit increases. This is because this is the peak water demand period for the maize fruit. The drought in the early stage hinders the water transportation to the fruit. After rehydration, the root absorbs water and transport most of the water to the aboveground parts, especially to meet the water demand of reproductive organs, ensure the cumulative maturity of fruit, and distribute most of the water to the fruit growth. In the MD **(** **Figure 4B** **)**, the WCDR of each component organ of summer maize was as follows: stem (38.79%) > fruit (27.25%) > leaf (25.02%) > root (8.94%). The WCDR of stem was significantly higher than that of the WCDR of the root, and the CS had a similar distribution rule at the same time. In RMD, the WCDR of the root increased, while the WCDR of the fruit and leaf decreased. This is because the root is close to the water source and gives priority to the root water supply. After the root absorbs water, it would try its best to compensate for its early drought water shortage; thus, the WCDR of the root would increase. In addition, this period is the ripening (dehydration period) of the maize fruit. The fruit water demand is reduced; thus, the WCDR of the fruit is reduced, while the WCDR of the leaf is reduced because of accelerated aging and withering due to drought in the early stage, the performance of the leaf is reduced, and the WCDR of the leaf is reduced.

### 3.2 Biomass allocation

#### 3.2.1 Biomass of component organs

**Figure 5:**
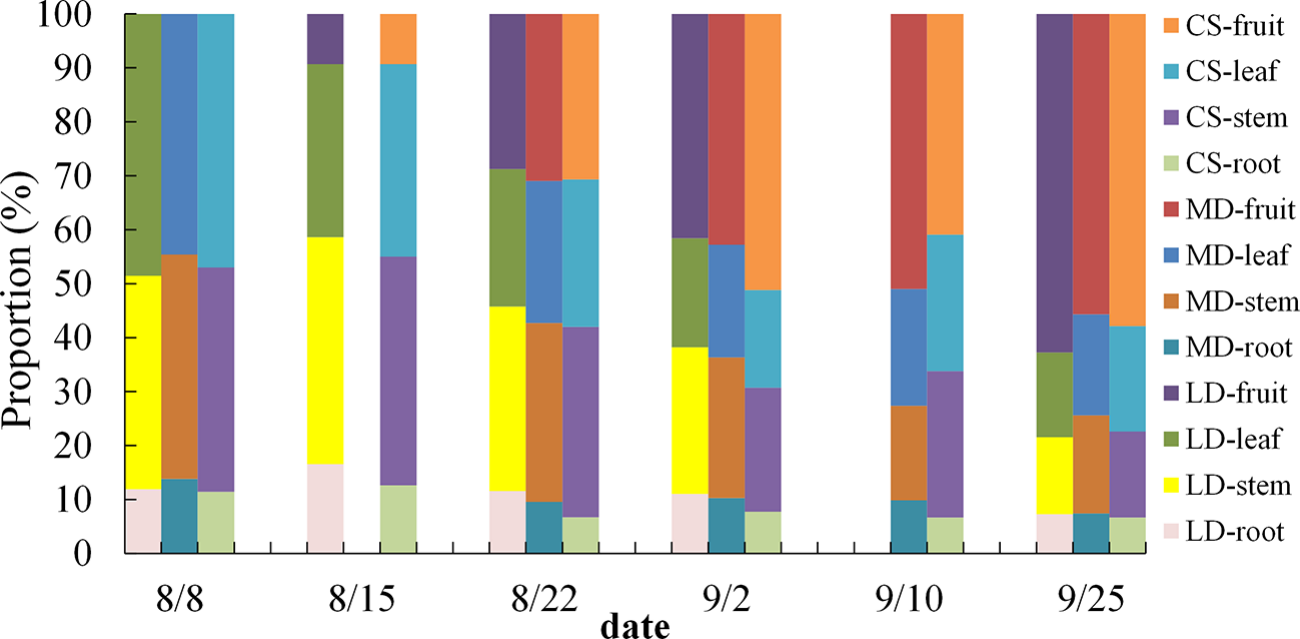
Dynamic change analysis of the biomass proportion of summer maize under different drought conditions.

The proportion of the biomass of summer maize varies with the growth period (**Figure 5**), calculated by **formula (5).** As a whole, the proportion of root biomass first increases and then decreases, accounting for 6.66%– 29.8% of the total biomass. The proportion of stem biomass (SB) and leaf biomass (LB) decreased slowly during the whole measurement period, and the proportion of SB accounted for 14.27%– 42.36%, the proportion of leaf accounts for 15.73%– 48.53%.The proportion of fruit biomass (FB) increased gradually throughout the measurement period, accounting for 0%–62.73% of the total biomass.

**Figure 6:**
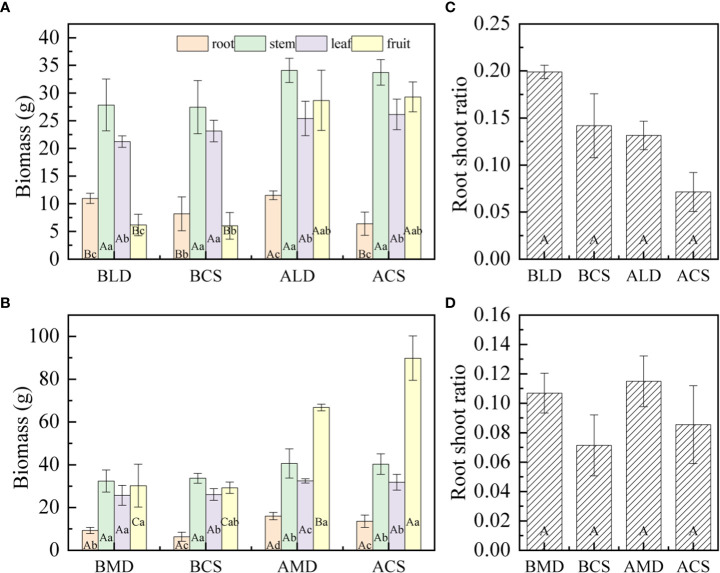
Comparative analysis of biomass and root shoot ratio of component organs in different drought and after rehydration. **(A, B)** are the comparison of biomass of each component organ of LD and MD, respectively; **(C, D)** are the comparison of root shoot ratio of organs in LD and MD, respectively. Error bars represent standard deviations (n =3), different capital letters indicate significant difference between groups (p<0.05), and different lowercase letters indicate significant difference between different organs in the same group (p<0.05). Note: BLD, BCS, ALD, and ACS and BMD, BCS AMD, and ACS have the same definitions as in **Figure 3**.

According to the analysis of the biomass of each component organ of summer maize drought and after rehydration **(** **Figures 6A, B** **)**, there is a significant difference between the biomass of component organs of summer maize. In LD, the biomass of each component organ of summer maize is as follows: stem (27.87 g) > leaf (21.27 g) > root (10.98 g) > fruit (6.18 g), and the SB is significantly higher than that of other component organs. In MD, the biomass of each component organ of summer maize is that stem (32.45 g) > fruit (30.3 g) > leaf (25.78 g) > root (9.35 g), and root biomass (RB) is significantly lower than that of other component organs.

In this study, drought would promote the accumulation of RB. The RB under LD and MD was 33.94% and 46.09% higher than the CS, respectively, with no significant difference. After rehydration, the RB increased by 5.16% and 71.48% compared with that of drought, respectively. The RB of RLD and RMD was 80.47% and 17.89% higher than that of the CS, respectively.

Different degrees of drought have different effects on SB. Under LD, it is conducive to the accumulation of SB. The SB of summer maize is 1.39% higher than that of the CS. Under MD, it would be adverse to the accumulation of SB. The SB is 3.80% lower than that of the CS. The SB increased after rehydration, the RLD and RMD increased by 22.37% and 25.48%, respectively, compared with that in drought. The SB was 1.09% and 0.87% higher than that of the CS in the same period in RLD and RMD, respectively. It can be seen that rehydration can effectively alleviate the inhibition of MD on SB accumulation.

The occurrence of LD and MD is not conducive to LB accumulation. Under LD and MD, the LB was 8.20% and 1.46% lower than the CS, respectively. The LB increased after rehydration; in RLD and RMD, it increased by 19.59% and 26.12%, respectively, compared with that in drought. It can be seen that after rehydration can compensate the early drought to a certain extent. Compared with that, the accumulation rate of LB in RMD is faster.

Drought is beneficial to the accumulation of FB. Under LD and MD, the FB was 2.49% and 3.35% higher than that of the CS, respectively. The FB increased after rehydration, the FB in LD and MD increased by 364.15% and 120.46% respectively compared with that in drought, but the FB in RLD and RMD was 2.10% and 25.68% lower than that in the CS, respectively.

According to the analysis of the root– shoot ratio of summer maize under different drought and after rehydration **(** **Figures 6C, D** **).** The root– shoot ratio of LD and MD was 0.20 and 0.11, respectively, which is higher than that of the CS, but there was no significant difference. The root– shoot ratio decreased in RLD, and the CS also had a similar rule in the same period. The root– shoot ratio increased slightly (little change) in RMD, and there was no significant difference with that in drought.

#### 3.2.2 Allometric growth model

**Figure 7:**
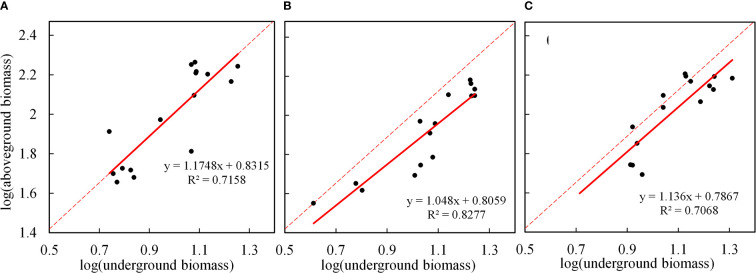
Allometric growth relationship between underground and aboveground biomass summer maize under different drought conditions. **(A–C)** are fitted for the allometric growth of the CS, LD, and MD, respectively. The red solid line represents the linear fitting under different drought scenarios, and the red dotted line represents the linear fitting of the CS.

The allometric growth of plants is an important manifestation of adaptation to heterogeneous habitats. The biomass allocation and growth relationship of plant component organs can effectively reveal the law of the allometric growth of plants. First, we found that by fitting and analyzing the CS data **(** **Figure 7A** **),** there was a very significant positive allometric growth relationship between the underground biomass and the aboveground biomass (P<0.001). That is, the aboveground part grew with the growth of the underground part, indicating that the underground part would affect the photosynthesis of the aboveground part while absorbing the growth of nutrients in the soil, and the aboveground biomass was also accumulating materials synchronously. To verify whether the model parameters of the CS are applicable to two different drought conditions, we analyzed R, R^2^, adjustment R^2^, and the RMSE **(** **Table 3** **)**, and found that the fitting accuracy was not good. Thus, we need to reformulate the model parameters to represent the allometric growth rule of the different drought scenarios. By fitting the data under two drought scenarios **(** **Figures 7B, C** **)**, the fitting accuracy is good, and different allometric exponents and coefficient are obtained. It is found that, under drought stress, the underground and aboveground parts also show positive allometric growth, CS (α=1.175) > MD (α=1.136) > LD (α=1.048). However, under drought, the allometric exponent is smaller than the CS. It also shows that, under drought, the growth of the aboveground part of maize would slow down with the growth of the underground part. Therefore, the growth trend of the aboveground part of maize with the growth of the underground part is slower than the CS. Compared with the two degrees of drought, the aboveground growth is slower in LD. The results in section 3.2.1 further support the accuracy of the model. Drought stress promoted the accumulation of RB (the RB under LD and MD was 33.94% and 46.09% higher than the CS, respectively) and inhibited the accumulation of LB (the LB under LD and MD was 8.20% and 1.46% lower than the CS, respectively).

**Table 3:** Fitting parameters and inspection indexes under different drought conditions.

Group	Different models	α	β	R	R^2^	Adjustment R^2^	RMSE	Optimal choice of model
CS	①	1.175	0.832	0.846	0.716	0.696	0.133	√
LD	①	1.175	0.832	0.649	0.421	0.376	0.183	×
	②	1.048	0.859	0.910	0.828	0.814	0.095	√
MD	①	1.175	0.832	0.683	0.466	0.425	0.130	×
	②	1.136	0.787	0.841	0.707	0.684	0.103	√

### 3.3 Analysis of correlation between water content and biomass of component organs

**Figure 8:**
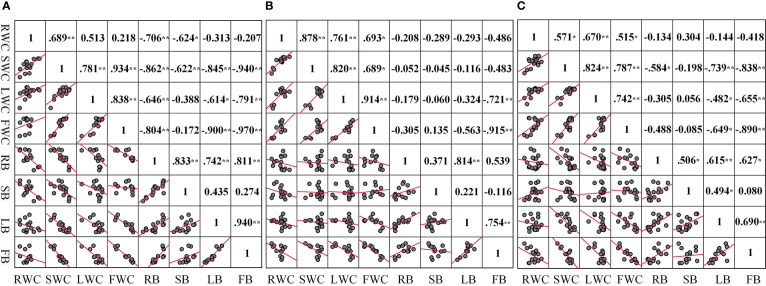
Correlation between the water content and biomass of each organ under different drought conditions **(A–C)** are the correlation between the water content and biomass of each component organ in LD, MD, and CS, respectively. *p<0.05; * *p< 0.01.

The correlation analysis was conducted on the water content and biomass of each component organ of summer maize under different drought conditions (**Figure 8** **)**. The water content of each component organ of summer maize has a negative correlation with the biomass of the corresponding component organ. The correlation coefficient between the FWC and the FB is high, and the degree of correlation is that LD (r =-0.97) > MD (r =-0.915) > CS (r =-0.89). Under LD, the correlation between the water content of each component organ and its corresponding component organ biomass is good; fruit (r =-0.97) > root (r =-0.706) > stem (r =-0.622) > leaf (r =-0.614). Under MD, the correlation coefficient between the water content of each component organ and its corresponding component organ biomass is that fruit (r =-0.915) > leaf (r =-0.324) > root (r =-0.208) > stem (r =-0.045).

The water content of each organ in the aboveground part of summer maize was positively correlated with that of the root. Among them, the correlation coefficient between the RWC and the SWC is high, and the degree of correlation is that MD (r =0.878) > LD (r =0.689) > CS (r =0.571). Under LD, the correlation coefficient between the RWC and the water content of each organ is that stem (r =0.689) > leaf (r =0.513) > fruit (r =0.218). Under MD, the correlation coefficient between the RWC and the water content of each organ is that stem (r =0.878) > leaf (r =0.761) > fruit (r =0.693).

There was a positive correlation between the biomass of organs and the biomass of roots in summer maize. Among them, the correlation coefficient between RB and LB is high, and the degree of correlation is that MD (r =0.814) > LD (r =0.742) > CS (r = 0.615). Under LD, the correlation coefficient between RB and the biomass of each organ was higher, which was stem (r =0.833) > fruit (r =0.811) > leaf (r =0.742). Under MD, the correlation coefficients between RB and the biomass of each organ are that leaf (r =0.814) > fruit (r =0.539) > stem (r = 0.371). These correlation coefficients further confirmed the adaptive strategy of the coordinated growth of water allocation and biomass allocation among various component organs of summer maize under drought stress.

### 3.4 Distribution characteristics of different component organs under different drought stress

**Figure 9:**
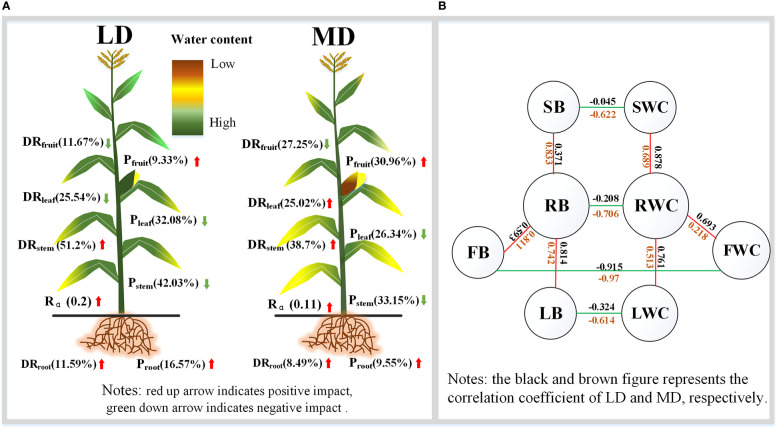
Characteristics of the resource allocation of summer maize organs under drought stress. **(A)** is the WCDR and proportion of biomass of each component organ under different drought conditions; **(B)** is the correlation between the water content and biomass of each component organ.

Through the analysis of the water content, WCDR, and biomass distribution of each component organ of summer maize under different drought stress, the distribution maps of each component organ of summer maize under different drought levels were drawn **(** **Figure 9** **)**. Under different levels of drought stress **(** **Figure 9A** **)**, there is a significant difference between the water content of each component organ. Drought would affect the water distribution ratio of each component organ. Under LD, the WCDR of the root and stem would increase, while the WCDR of the leaf and fruit would decrease. Under MD, the WCDR of the root, stem, and leaf would increase, while the WCDR of the fruit would decrease. This also indicates that crops would give priority to allocate water to component organs closer to the water source during drought. Summer maize would also adjust and actively adapt to drought in terms of biomass accumulation. Whether it is LD or MD, the RB and FB of summer maize increases, while the SB and LB decreases, making the root– shoot ratio increase. Thus, the allometric exponent of underground– aboveground biomass fitting is smaller than that of the CS. The regulation of the underground and aboveground parts of summer maize is inseparable **(** **Figure 9B** **)**. The water content of each component organ of summer maize is negatively correlated with its corresponding biomass. The RWC is positively correlated with the SWC, LWC, and FWC. Similarly, RB is also positively correlated with SB, LB, and FB. It shows that each component organ of summer maize has a closer coordination in the distribution of water and biomass. This also provides a theoretical study on the mechanism of the mutual regulation among the component organs.

## 4 Discussion

### 4.1 Water content distribution

The distribution of water in plants can directly reflect the pattern of water acquisition and utilization among plant components and organs. In this study (**Figure 2** **)**, the RWC of summer maize showed a decrease first. Then, it increased and then decreased during the measurement period, and the SWC, LWC, and FWC gradually decreased with the growth period. During drought, the general trend of the water content in component organs is that the water content decreases gradually with the increase of days without rainfall, which is consistent with some scholars’ conclusion (Wang et al., 2015; Abdalla and Ahmed, 2021). The LWC is higher than the CS during drought, the reason may be that with the leaf as the main photosynthesis site, the water retention of maize is more obvious under drought stress. By reducing the leaf area and closing their stomata, transpiration consumption is reduced to maintain the LWC, so as to conduct photosynthesis (Sade et al., 2012). Thus, the LWC is higher than that of the CS. This is consistent with some studies; when the drought is in a certain range, the LWC would increase (Ma et al., 2021).

There is a significant difference in the water content of the component organs of summer maize, and the difference changes with the growth period. In this study (**Figure 3** **)**, in LD, the water content of each component organ is as follows: fruit > stem > leaf > root. The SWC and FWC were significantly higher than that of the LWC and RWC, which was similar to the CS at the same period. However, In MD, the water content of each component organ is that stem > leaf > root > fruit; the SWC was significantly higher than the RWC and FWC. The difference of the water content of each component organ is different from the CS. The difference in the water content of each component organ is mainly due to the large range of changes in the FWC. The main reason for the difference in LD is the difference of the water content of each component organ caused by the different composition and content of each component organ; it is less affected by drought. Generally, the root and stem contain more cellulose, and the fruit contains more sugar and starch. Due to the sampling date being August 15 in LD, which is just at the beginning of the ear stage of the fruit, the fruit component organs are delicate, growing vigorously, and the water content is generally high. This is consistent with some results: the water content of the vigorous component organs is higher than that of the aged component organs, the water content of the upper component organs is higher than that of the lower component organs, and the water content of the meristem and conducting tissues is higher than that of the epidermis, cortex, and other tissues (Shinn and Lemon, 1968). There are two main reasons for the differences in the water content of component organs caused by MD. One is that the sampling time of MD is August 22; at this time, the fruit had been growing for a period of time, not as delicate as at the beginning of heading, and the FWC is lower than before. The second is that the water content of each organ is different due to drought. Drought would make the RWC, SWC, and FWC lower than the CS, while the LWC is higher than the CS. Therefore, the difference of the water content of the component organ during MD is not consistent with that of the CS. This is also consistent with the conclusions of some studies; in different environments, the water content of different component organs of the same plant has a large difference (Sternberg and Shoshany, 2001).

In this study (**Figure 4** **)**, the WCDR of the root in different treatment groups was the lowest, and the WCDR of the stem was the highest. Although the WCDR of root was the lowest among all component organs, it was higher than the CS at the same period under drought, while the WCDR of the fruit was lower than that of the CS at the same period. This also shows that, under drought, more water would be allocated to the root to improve the water absorption capacity, so as to provide enough water for the component organs above the ground, which is related to the physiological function of the root (Prince et al., 2022; Sandar et al., 2022). The summer maize fruit is the reproductive organ, and its physiological activity is the most vigorous component organ at this stage. The effect of drought leads to the reduction of the WCDR of the fruit, which is because the fruit is far away from the water source and the root absorbs water and then transmits it to the fruit, there is a certain height between the root and the fruit that would hinder the water transportation (Scholander et al., 1965). The maize gives priority to allocate water to the component organs closer to the water source (priority: root > stem > leaf > fruit). Drought reduces the water transportation capacity, thereby reducing the WCDR of the fruit. Drought reduces the water transport capacity from the root to the fruit; thus, the WCDR of the fruit is reduced.

### 4.2 Biomass allocation

The proportion of the biomass of each component organ of a plant represents the distribution proportion of assimilation products to different component organs and the coordination relationship of each component organ in the growth process (Yang et al., 2021). In this study (**Figure 5** **)**, the proportion of the RB of summer maize increased first and then decreased, the proportion of SB and LB decreased slowly throughout the measurement period, and the proportion of FB increased gradually throughout the measurement period. The biomass of each component organ of summer maize is that (**Figure 6** **)** in LD, stem (27.87 g) > leaf (21.27 g) > root (10.98 g) > fruit (6.18 g). In MD, stem (32.45 g) > fruit (30.3 g) > leaf (25.78 g) > root (9.35 g). In the same period, the CS had similar laws. The stem is a huge energy reservoir and regulator of a plant; in general, the biomass allocated is large (Xu et al., 2020). In this study, drought increased RB, decreased LB, and increased the root– shoot ratio; this is consistent with some research results (Madhav et al., 2017; Sofi et al., 2017; Gao et al., 2022). Under drought stress, summer maize makes corresponding changes to adapt to the environment at this time. The first is to reduce evaporation. When the water is limited, summer maize itself would give priority to divide water to roots to form larger roots; thus, RB increases and then transports more nutrients to the ground as much as possible. The transpiration of summer maize is mainly carried out by the leaves. The leaves would wither and curl in order to keep water (Ye et al., 2020). They would turn yellow and fall off in severe drought, which would inhibit the growth and reduce the LB.

Plant growth is the result of balancing various resources allocated by plants to the root, stem, leaf, and other component organs (Reich et al., 1998; Xu et al., 2015). There are significant correlations and allometric growth relationships between crop component organs, which reflect the cooperative growth strategy of various tissues (Yuan et al., 2018). In this study (**Figure 8** **)**, the water content of each component organ of summer maize has a negative correlation with its corresponding biomass. This is because the overall trend of the water content of each component organ decreases with the increase of growth time, while the biomass gradually increases with the increase of time; thus, there is a negative correlation. The RWC of summer maize is positively correlated with the water content of each component organ, and RB is also positively correlated with the biomass of each component organ, which reflects that the underground and aboveground parts of summer maize are inseparable, and the underground and aboveground component organs need to maintain a relatively stable coordinated growth. According to the biomass allocation theory put forward by some scholars (Enquist and Niklas, 2002), the underground and aboveground parts of plants under a non- stress environment show the isokinetic growth, but, under the water shortage or other environments, each component organ of plants is likely to change into allometric growth because plants often adapt to a specific growth environment by adjusting their own resource allocation during the growth and development process, so as to achieve the goal of coordinated growth and reproduction (Boutraa, 2010). In this study (**Figure 7** **)**, the underground biomass and aboveground biomass of summer maize under different conditions have a very significant positive allometric growth relationship, that is, the aboveground part grows with the underground part. However, under drought, the allometric exponent is smaller than the CS. It also indicates that the growth of the aboveground part is slower than that of the CS with the growth of the underground part. From the section 3.2.1 results, we can find out that, under drought stress, the root would grow preferentially, and RB increased while LB decreased. Therefore, the allometric exponent is smaller than the CS under drought, This biomass allocation pattern can optimize resource utilization to ensure maximum growth (Rigoberto et al., 2004).

## 5 Conclusion

Our research focused on the coordinated growth strategy of the water content and biomass allocation of each component organ of summer maize under drought stress. The results showed that under drought stress, summer maize would preferentially distribute water to the component organs close to the water source; thus, the WCDR of the root and stem increased 1.83%– 2.35% and 0.52%– 1.40%, respectively. Drought would promote BR and FB while inhibiting the SB and LB, so as to increase the root– shoot ratio. We also verified this by establishing the allometric growth model. It was found that under drought stress, the allometric exponent α under drought stress is lower than that of the control group in shed. It also shows that existing estimates of the impact of climate change on food production may be underestimated. The results of this study basically verified our hypothesis. Further research can add drought experiments of different grades and measure the distribution characteristics of enzymes, proteins, and so on among component organs. Therefore, the coordination and adaptability of each component organ under different drought stresses can be further explored, providing a theory for improving water use efficiency.

## Data availability statement

The original contributions presented in the study are included in the article/**Supplementary Material**. Further inquiries can be directed to the corresponding author.

## Author contributions

SY and LJ collected basic data. SY performed the statistical analysis and wrote the first draft of the manuscript. BW provided the working concept. The manuscript was critically revised by BW, and WB provided modification suggestions. All authors contributed to this article and approved this version for submission.
